# The Power of the Picture: How Narrative Film Captures Attention and Disrupts Goal Pursuit

**DOI:** 10.1371/journal.pone.0144493

**Published:** 2015-12-10

**Authors:** Anna-Lisa Cohen, Elliot Shavalian, Moshe Rube

**Affiliations:** Yeshiva University, New York, NY, United States of America; University of Bologna, ITALY

## Abstract

*Narrative transportation* is described as a state of detachment that arises when one becomes immersed in the narrative of a story. Participants viewed either an intact version of an engaging 20 min film, “Bang You’re Dead!,” (1961) by Alfred Hitchcock (*contiguous condition*), or a version of the same film with scenes presented out of order (*noncontiguous condition*). In this latter condition, the individual scenes were intact but were presented out of chronological order. Participants were told a cover story that we were interested in the amount of gun violence depicted in films. Both groups were given the goal to remember to lift their hand every time they heard the word “gun” spoken during the film. Results revealed that participants were significantly less likely to remember to execute their goal in the contiguous condition, presumably because this narrative transported viewers’ attention and thereby “hijacked” processing resources away from internal goals.

## Introduction

The power of stories to transport the audience represents a fundamental part of human experience. Gerrig [1] was the first to coin the term *narrative transportation* in the context of written literature. Narrative transportation occurs when an individual experiences the feeling of entering the world evoked by a narrative because of empathy for story characters and imagination of the story plot [2]. It is described as a state of detachment from the world, as though one is being carried away by the story. Much has been written in film literature about how techniques of cinema function to engage the viewer. Only quite recently [3] have scientists considered cinema as a topic for empirical investigation. Researchers describe narrative transportation as a state of simulation [4]. For example, they suggest that readers of novels, filmgoers, and theatergoers all undergo a simulation of events when they experience what feels like genuine sorrow when a beloved hero dies, despite the fact that events depicted in the narrative are not real.

Not all stories are equivalent in their ability to transport the reader or viewer. For example, researchers have explored the extent to which brain activity differs across participants during film viewing and found that films varied substantially in their ability to engage the viewer [5]. Participants viewed films while undergoing functional magnetic resonance imaging (fMRI). Intersubject correlation (ISC) measures the similarities in brain activity across viewers. Movies with a high ISC are highly engaging and trigger similar emotional and cognitive responses from viewers leading to higher intersubject synchronization. Results of this study revealed especially high levels of inter-subject correlations in certain films (e.g., a film by Alfred Hitchcock) compared to others [5]. These results provided neuroscientific evidence for Hitchcock’s reputed ability to artfully engage and control viewers’ attention. As Shimamura and colleagues observed, when filmmakers are successful they are able to guide the viewers’ attention to points in a scene [6]. Work by other researchers [7] showed consistency in gaze patterns in individuals while they watched clips of feature films. At certain points during film viewing, eyetracking data showed that virtually all participants were fixated at the same point on the screen at the same time. This phenomenon of gaze attraction has been termed *attentional synchrony* [8]. Results from eyetracking and fMRI studies show how narrative films can guide our attention so effectively that virtually everyone in the theater is attuned to the same perceptual features [6].

The experience of narrative transportation is similar to other engaging experiences such as absorption [9] and flow [10]. However, there are subtle but critical differences between narrative transportation and these other experiences [2]. For example, absorption refers to a dispositional trait that can be low or high in individuals and describes a general tendency to become immersed in experiences such as fantasy and mental imagery. Transportation, by contrast, is an engaging experience that is temporary and occurs only in response to a story narrative. Flow is a more general construct in which an individual experiences complete and total focus on a specific activity. Narrative transportation involves empathy with story characters and mental imagery, which do not necessarily occur in flow experiences [2].

A consumer of these narrative experiences constructs a mental model by incorporating information from the narrative along with knowledge that he or she already possesses from personal experience [11]. The concept of mental models [12] is similar to situation models [13] [14] which is the term used in the reading comprehension literature. A situation model refers to the mental representation that a reader constructs of the events described in a narrative. This idea of narrative processing places the audience member as an active participant because he/she is dynamically creating the story as the narrative unfolds [15] [1]. Research shows that as we construct situation models we infer causality and the goals of the protagonist [14]. Thus, if a protagonist has a goal that has not yet been accomplished, that goal is more accessible to the reader than a goal that was just accomplished by the protagonist. In line with this prediction, goals yet to be accomplished by the protagonist were recognized more quickly than goals that were just accomplished [16]. One can think of suspense in films as situations in which the goal of a protagonist takes on a more heightened value. That is, it may be that suspense heightens the importance of a perceived goal. Indeed, Bezdek and colleagues [17] tested the hypothesis that, in moments when suspense increases, narrative transportation will produce a changing pattern of activity in brain regions involved in early visual processing. They used fMRI to show that spatially peripheral stimuli received suppressed early visual processing when suspense increased in narrative film scenes. Participants viewed film excerpts that incorporated high suspense scenes while checkerboards flashed continuously in the visual periphery. Results supported their hypothesis that in moments of increased threats to characters, there was a corresponding increase in activity to central visual regions and suppression of activity in peripheral visual regions [17].

### The Present Study

In the current study, we gave participants a simple goal: to remember to lift their hand every time they heard the word “gun” spoken during the film. In one condition, the film was presented in its intact form while, in the other condition, the film was presented in a noncontiguous form (i.e., with the scenes out of sequence). The film “Bang! You’re Dead” by Alfred Hitchcock (1961) has been shown to be highly engaging as reflected by high inter-subject correlations [5]. Goals that have yet to be accomplished by a story protagonist become more salient to the reader [16]. The activation of alternative goals may pull resources away from the focal goal, and hence undermine goal attainment [18]. Therefore, we predicted that, as the story progressed and suspense increased, the goals of the protagonist would take on greater value. In the contiguous condition, identification with story characters could be built in a gradual and natural way leading to a corresponding increase in concern and empathy for those characters. As a result, we predicted that in the contiguous condition attention would increasingly be captured by the film leading to narrative transportation and neglect of participant’s own goal. The noncontiguous film condition served as a perfectly matched control as we used the identical film but presented it with the scenes out of order.

## Method

### Participants

A total of 50 Yeshiva University male undergraduate students volunteered to participate in the experiment in exchange for course credit as a part of their psychology course or $5.00. Each participant was tested individually in sessions that lasted ~30 min.

### Materials and Procedure

The IRB at the Albert Einstein College of Medicine which is the IRB that reviews all Yeshiva University studies approved the study (IRB #:2013–2742; Reference #:001649). After signing the informed consent form, participants were instructed to read the instructions for the experiment. Instructions were presented on the computer screen and participants were given a cover story that we were interested in the amount of gun violence depicted in film. The instructions were as follows: “As you may be aware, there have been many shootings in the United States over the past few years and we are interested in the portrayal of violence in popular culture. In this task, you will be watching a short film and we will ask you questions at the end. One thing we want you to remember to do is raise your index finger in the air every time you hear the word “gun” spoken at any point during the video. We will not be reminding you of this instruction once the videos begin. Once you have understood the directions, notify your experimenter.”

Participants were randomly assigned to one of two conditions, yielding 25 participants in each condition. A power analysis revealed sufficient power (0.80) to detect a medium sized effect between conditions; therefore, we stopped testing at 25 participants per condition [19]. After reading the instructions, participants were informed that they would view a short film with either intact scenes (contiguous condition) or out of order scenes (noncontiguous condition) but that, in either case, their goal was to remember to raise their hand every time they heard the word “gun” spoken in the film. Once participants showed comprehension, the film began with no further reminders of these instructions. The film we used was a highly engaging 20 min film by Alfred Hitchcock titled “Bang! You’re Dead!” (1961). The synopsis of this film from IMDb (http://www.imdb.com/) is as follows “Rick Sheffield visits his brother and sister-in-law after a lengthy absence living in Africa. His nephew Jackie [who is 5 years old] unpacks his suitcase and finds a revolver. Jackie and his friends are always playing with their toy guns and Jackie goes around town, pointing the gun and pulling the trigger, oblivious to the fact that there is a live round in the chamber. When his parents and uncle realize he has the gun, they set off on a frantic search but not before he fires at someone” (Appendix A in S1 File).

Participants in the contiguous condition viewed the film in its unscrambled form. In the noncontiguous condition, we disrupted temporal continuity by shuffling the scenes so that they were presented out of sequence. Each individual scene was intact but the order in which the scenes were presented was out of order. The intact version of the film could be separated into 27 separate scenes and some of the scenes were broken up into sub-scenes. The noncontiguous film comprised the same 27 scenes and sub-scenes, but rearranged and presented out of order (Appendices B and C in S1 File). Therefore, participants in each condition viewed the identical film with the same goal to raise their hand every time they heard the word “gun” spoken. Goal responses of participants were recorded discreetly by the experimenter who was seated out of view behind the participant. In both conditions, the word “gun” was spoken 7 times throughout the film at similar time points.

## Results and Discussion

**Fig 1:**
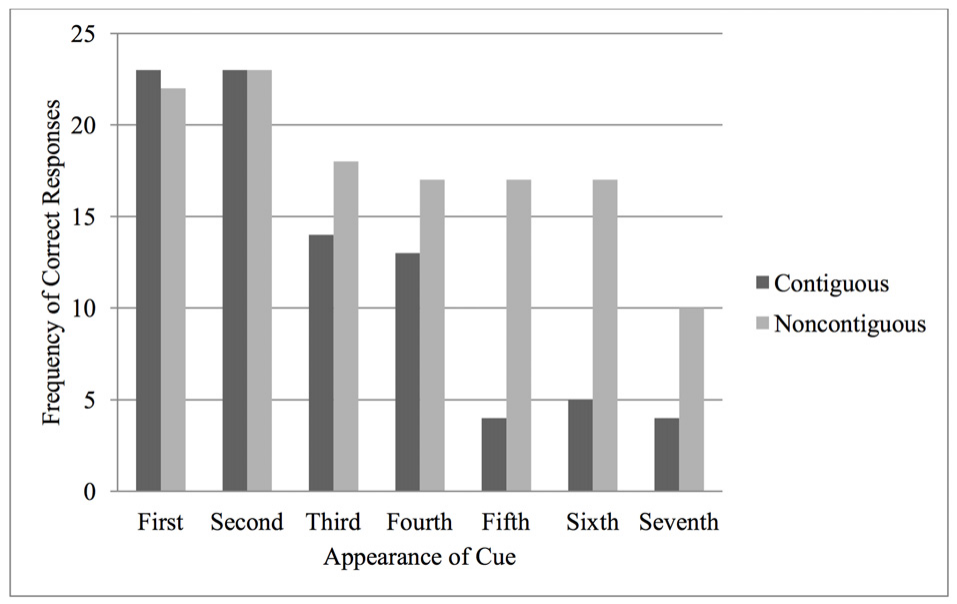
Frequency of correctly responding to the word “gun” as a function of condition (contiguous, noncontiguous) and appearance of cue.

An alpha level of .05 was used in all analyses unless otherwise specified. Results revealed a significant difference between goal execution in the contiguous and noncontiguous conditions *t*(48) = -3.11, *p* = .003, Cohen’s *d* = 0.88. Participants in the noncontiguous condition remembered to respond to the word “gun” significantly more times (*M* = 5.0 out of 7, *SD* = 2.0) than participants in the contiguous condition (*M* = 3.4 out of 7, *SD* = 1.6)^1^. To further examine performance across the 7 time points, we analyzed the likelihood of goal execution as a function of time and condition using a logistic regression model with generalized estimating equation (GEE) standard errors, which take into account the fact that time points were repeated. Results revealed a decline across time points in the noncontiguous condition (β = -.37, *SE* = .09, *p* = .001) but the decline was much larger in the contiguous condition (interaction β = -.42, *SE* = .17, *p* = .01; slope (-.37+-.42) = -.79). Inspection of Fig 1 shows that performance in the first 3 to 4 time points was similar across the two conditions, while performance in the 5^th^ and later trials declined more so for those in the contiguous condition. We conducted a binary logistic regression at each of the seven time points to examine whether there was a significant difference in the frequency of correct responses as a function of condition. There was no significant effect of condition at time points 1 through 4 (*p* = .638, *p* = 1.00, *p* = .242, *p* = .251). However, there was a significant difference at time points 5 and 6, (both *p*s = .001) and a marginal effect at time point 7 (*p* = .066).

Results revealed that participants were significantly less likely to remember to execute their goal in the contiguous condition than in the noncontiguous condition. Although participants in both conditions remembered their goal less and less as the film progressed, the decline was significantly greater in the contiguous condition. Data from Hasson et al. [5] showed that in highly engaging films, such as Sergio Leone’s “The Good the Bad and the Ugly,” fMRI and eye-tracking data revealed that viewers were guided to attend to the actions of the protagonist in similar ways. However, this was not the case for less engaging films, such as surveillance video or films with the scenes presented out of sequence [20]. Research has shown that if a filmmaker fails to direct viewers’ attention, each viewer will attend to and process different information at each moment in time, which will subsequently increase variability in brain responses across viewers [5]. Given that participants in the current experiment viewed the identical film in the two conditions with the only difference being the order of scenes; it is likely that the noncontiguous condition led to more variability in participants’ attention to the film. This may have allowed participants a chance to more easily disengage from processing the narrative in the noncontiguous condition to execute their goal.

Our results provide behavioral evidence that are very much in line with those of Bezdek and colleagues [17] who provided the first neural evidence that attention narrows in early visual processing regions as suspense is heightened. In the film “Bang! You’re Dead”, the main character, a five year old named Jackie, walks around town with his uncle’s loaded gun pointing it at people, thinking it is a toy. His parents set off on a frantic search to find him. As the story progresses, an enduring feeling of dread and foreboding develops. The viewer witnesses the parents’ panic as they set off on a search to find their child. Although we did not include a direct measure of suspense in this study, Bezdek and colleagues [17] suggest that suspense is one of the factors linked to increased transportation and it arises when potential threats to characters become salient and is characterized as a mixture of fear for a negative outcome and hope for a positive outcome. As Van Laer et al. [2] state (see also [21]), the state of narrative transportation leads to the world of origin becoming partially inaccessible. According to “suture theory” [22], the audience “stitches” themselves into the film by relating to characters or world views expressed in the film. Thus, in the contiguous condition, identification with story characters was built in a gradual and natural way leading to a corresponding increase in concern and empathy when those characters were threatened. Kuhl [23] has argued that progress toward goals involves both the active pursuit of a chosen focal goal and the inhibition of alternative goals that might come to mind. As the importance of a character’s goal increased in significance in the narrative (i.e., mother’s frantic attempt to find her son), attention narrowed to focus on relevant details of the story leading to neglect of participants’ own goal.

The goal that we gave participants was akin to an “open goal” [24] or *prospective memory* [25], that is, a goal or intention that has been formed but for which the associated task has not yet been completed. In this study, the intention to respond to “gun” had to be maintained across the span of a 20 minute film, with no reminders once the film began. Therefore, some proportion of processing resources needed to be set aside to monitor this goal [26].

Proponents of a working memory load model [27] might have predicted that reconstructing the story plot from scenes in the noncontiguous condition was an effortful task and, thus, would have expected worse goal performance in this condition. However, this was not the case. Based on our debriefing, participants in the noncontiguous condition were able to understand the plot even with scenes out of order. This may explain why there was a decline in goal maintenance in the noncontiguous condition (albeit not as pronounced as in the contiguous version). That is, there might have been some narrative transportation operating as participants pieced together the scenes and began to understand the plot. Participants in the noncontiguous version may have been able to resist being totally captured by the plot because they were less emotionally engaged. Given that the story was essentially understood in both conditions, our data suggest that cinematic techniques designed to evoke dramatic tension and suspense in the contiguous condition may have been rendered less effective when presented in a noncontiguous sequence. If participants experienced more narrative transportation in the contiguous condition, then they might have felt more emotionally invested in the story characters and the outcome. Kron and colleagues [28] show that emotion is a mental phenomenon which requires processing resources; thus, as emotional engagement intensifies there may be correspondingly fewer resources left over to maintain ongoing activities such as goal monitoring. These data speak to the importance of the intended temporal sequence and chronological flow of events and their ability to capture viewers’ attention. In a well known study [29], observers were “blind” to a gorilla entering a room when they were given the task of counting the number of times players passed a ball to each other. An implication of the above study and our own may be that events that are sequenced in a dynamic and coherent way, make disengaging (whether it is to notice a gorilla or remember one’s goal) much more difficult. This sequencing may create the perfect platform for filmmakers to engage viewers and manipulate attention. In line with these ideas, Shimamura et al. [6] showed that when subjects were immersed in the plot of a movie with their attentional focus riveted to the screen, then they were oblivious to the innumerable cuts and edits employed by directors, which might have otherwise resulted in a jarring visual experience.

To conclude, we introduced a new paradigm for measuring the effect of film on viewers’ attention and it also provided an opportunity to examine prospective memory (remembering a goal or intention) in a more realistic and natural setting. The contiguous and noncontiguous conditions were closely matched containing the exact same content but with scenes presented in different orders. Based on our results, it seems that altering the order of scenes in the noncontiguous condition disrupted narrative transportation allowing participants to maintain their goal more easily. This translates to everyday situations in which we might have an important goal (e.g., to take dinner out of the oven) but we become engrossed in a highly engaging story. Our study shows how competing motives (viewing a narrative and maintaining one’s goal) involve a type of tension. As attention is captured by the film, we lose subjectivity and the focus on our own goals is replaced by focus on the goals portrayed in the film.

## Supporting Information

S1 File Scene breakdown for contiguous version of the film (Appendix A). Scene breakdown for contiguous condition showing where the cue “gun” appeared (Appendix B). Scene breakdown for noncontiguous condition showing where the cue “gun” appeared (Appendix C). (DOCX)
