# Supplementary material for: The Power of the Picture: How Narrative Film Captures Attention and Disrupts Goal Pursuit
Source: PLoS One. 2015 Dec 10;10(12):e0144493. doi: 10.1371/journal.pone.0144493 (PMC4675523; doi:10.1371/journal.pone.0144493)
Supplement: S1 File — (DOCX) [file pone.0144493.s001.docx]

**Supporting Information**

**S1. Appendix A.**

Scene 1 - Intro, children are playing with toy guns in front yard of Jackie’s house

Scene 2 – Dad and uncle pull up in car and mother comes out to greet them

Scene 3 - Family in living room chatting

Scene 4 – Father and mother in living room

Scene 5 - Uncle begins unpacking his suitcase with Jackie in upstairs bedroom

Scene 6 – Jackie’s father comes upstairs and asks uncle to come downstairs

Scene 7 – Jackie continues unpacking for uncle and discovers real gun and puts it in his pocket

Scene 8 - Family is in living room/mother is on phone/ Jackie leaves the house

Scene 9 - Jackie pretends to “hold up” mailman with gun then continues walking through town

Scene 10 - Uncle realizes that his gun is missing and that Jackie has taken it

Scene 11 - Jackie walks to supermarket and amuses himself playing with the gun

Scene 12 - Mother is back at home calling friends trying to find her son

Scene 13 - Uncle asks neighborhood kids if they have seen Jackie

Scene 14 - Jackie is at store riding on coin operated horse ride

Scene 15 - Jackie walks into the supermarket

Scene 16 – Mother walks in the store frantic but she can’t find him

Scene 17 – Store is very busy and everyone ignores the mother

Scene 18 - Mother continues looking around in store for her son

Scene 19 – Manager makes announcement on intercom that Jackie should report to the office

Scene 20 - Jackie runs out of the store and doesn’t hear the announcement

Scene 21 – Sales lady says that she saw her son a few minutes ago

Scene 22 – Mother grabs intercom speaker and makes frantic announcement

Scene 23 – Mother, father, uncle are in parking lot searching for Jackie

Scene 24 - Jackie walks home oblivious to the fact that everyone is looking for him

Scene 25 – Jackie interacts with housekeeper Cleo who has just arrived

Scene 26 - Family pulls up to the house

Scene 27 - Jackie fires shot at Cleo just as the family rushes into the house

**S1 Appendix B.**

| Scene 1.1 | | |
| --- | --- | --- |
| Scene 1.2 | | |
| Scene 1.3 (Cue) | | |
|  |  |  |
| Scene 2.1 | | |
| Scene 2.2 | | |
| Scene 2.3 | | |
| Scene 2.4 | | |
| Scene 2.5 | | |
| Scene 2.6 | | |
| Scene 2.7  Scene 2.8 | | |
|  |  |  |
| Scene 3.1 (Cue) | | |
| Scene 3.2 | | |
| Scene 3.3 | | |
| Scene 3.4 | | |
| Scene 3.5 | | |
| Scene 3.6 | | |
|  |  |  |
| Scene 4 | | |
|  |  |  |
| Scene 5 | | |
|  |  |  |
| Scene 6 | | |
|  |  |  |
| Scene 7.1 | | |
| Scene 7.2 | | |
| Scene 7.3 | | |
| Scene 7.4 | | |
| Scene 7.5 | | |
| Scene 7.6 | | |
| Scene 7.7 | | |
| Scene 7.8 | | |
| Scene 7.9 | | |
| Scene 7.10 | | |
| Scene 7.11 | | |
| Scene 7.12 | | |
| Scene 7.13 | | |
| Scene 7.14 | | |
| Scene 7.15 | | |
| Scene 7.16 | | |
|  |  |  |
| Scene 8.1 | | |
| Scene 8.2 | | |
| Scene 8.3 | | |
|  |  |  |
| Scene 9.1 | | |
| Scene 9.2 | | |
| Scene 9.3 | | |
| Scene 9.4 | | |
|  |  |  |
| Scene 10.1 | | |
| Scene 10.2 | | |
| Scene 10.3 | | |
| Scene 10.4 | | |
| Scene 10.5 | | |
| Scene 10.6 | | |
| Scene 10.7 | | |
| Scene 10.8 | | |
| Scene 10.9 | | |
| Scene 10.10 | | |
| Scene 10.11 | | |
|  |  |  |
| Scene 11 | | |
|  |  |  |
| Scene 12.1 | | |
| Scene 12.2 | | |
| Scene 12.3 | | |
| Scene 12.4 | | |
|  |  |  |
| Scene 13.1 | | |
| Scene 13.2 (Cue) | | |
| Scene 13.3 (Cue)  Scene 13.4 | | |
|  |  |  |
| Scene 14.1 | | |
| Scene 14.2 | | |
| Scene 14.3 | | |
| Scene 14.4 | | |
| Scene 14.5 | | |
| Scene 14.6 | | |
| Scene 14.7 | | |
| Scene 14.8 | | |
| Scene 14.9 | | |
|  |  |  |
| Scene 15 | | |
|  |  |  |
| Scene 16.1 | | |
| Scene 16.2 | | |
| Scene 16.3 | | |
|  |  |  |
| Scene 17.1 | | |
| Scene 17.2 | | |
|  |  |  |
| Scene 18.1 | | |
| Scene 18.2 | | |
| Scene 18.3 | | |
| Scene 18.4 | | |
|  |  |  |
| Scene 19.1 | | |
| Scene 19.2 | | |
|  |  |  |
| Scene 20 | | |
|  |  |  |
| Scene 21.1 | | |
| Scene 21.2 | | |
| Scene 21.3 (Cue) | | |
|  |  |  |
| Scene 22.1 | | |
| Scene 22.2 | | |
| Scene 22.3 | | |
|  |  |  |
| Scene 23 | | |
|  |  |  |
| Scene 24 | | |
|  |  |  |
| Scene 25.1 | | |
| Scene 25.2 | | |
| Scene 25.3 | | |
| Scene 25.4 (Cue) | | |
|  |  |  |
| Scene 26 | | |
|  |  |  |
| Scene 27 (Cue) | | |
|  |  |  |
|  | | |

|  |  |  |
| --- | --- | --- |
|  |  |  |
|  |  |  |
|  |  |  |
|  |  |  |
|  |  |  |
|  |  |  |
|  |  |  |
|  |  |  |
|  |  |  |
|  |  |  |
|  |  |  |
|  |  |  |
|  |  |  |
|  |  |  |
|  |  |  |
|  |  |  |
|  |  |  |
|  |  |  |
|  |  |  |
|  |  |  |
|  |  |  |
|  |  |  |
|  |  |  |
|  |  |  |
|  |  |  |
|  |  |  |
|  |  |  |
|  |  |  |

| **S1 Appendix C.**   \| Scene 2.8 \| \| --- \| \| Scene 2.6 \| \| Scene 14.9 \| \| Scene 2.7 \| \| Scene 22.2 \| \| Scene 25.4 (Cue) \| \| Scene 7.15 \| \| Scene 5.1 \| \| Scene 7.16 \| \| Scene 10.11 \| \| Scene 16.3 \| \| Scene 9.4 \| \| Scene 10.10 \| \| Scene 7.1 \| \| Scene 11.1 \| \| Scene 21.1 \| \| Scene 10.1 \| \| Scene 18.3 \| \| Scene 12.1 \| \| Scene 18.1 \| \| Scene 14.4 \| \| Scene 3.1 (Cue) \| \| Scene 2.1 \| \| Scene 7.9 \| \| Scene 12.3 \| \| Scene 14.1 \| \| Scene 2.3 \| \| Scene 9.3 \| \| Scene 7.7 \| \| Scene 8.2 \| \| Scene 14.2 \| \| Scene 3.2 \| \| Scene 16.2 \| \| Scene 7.8 \| \| Scene 16.1 \| \| Scene 2.2 \| \| Scene 9.1 \| \| Scene 10.5 \| \| Scene 7.5 \| \| Scene 20.1 \| \| Scene 7.3 \| \| Scene 14.3 \| \| Scene 3.4 \| \| Scene 14.5 \| \| Scene 10.4 \| \| Scene 3.3 \| \| Scene 10.6 \| \| Scene 7.12 \| \| Scene 19.2 \| \| Scene 14.6 \| \| Scene 14.7 \| \| Scene 3.6 \| \| Scene 3.5 \| \| Scene 10.9 \| \| Scene 17.2 \| \| Scene 4.1 \| \| Scene 25.1 \| \| Scene 7.10 \| \| Scene 7.4 \| \| Scene 21.3 (Cue) \| \| Scene 17.1 \| \| Scene 7.13 \| \| Scene 9.2 \| \| Scene 2.5 \| \| Scene 26.1 \| \| Scene 1.2 (Cue) \| \| Scene 10.2 \| \| Scene 1.3 \| \| Scene 7.14 \| \| Scene 18.4 \| \| Scene 1.1 \| \| Scene 2.4 \| \| Scene 8.1 \| \| Scene 8.3 \| \| Scene 14.8 \| \| Scene 19.1 \| \| Scene 22.1 \| \| Scene 10.3 \| \| Scene 13.1 \| \| Scene 24.1 \| \| Scene 12.4 \| \| Scene 7.6 \| \| Scene 13.3 (Cue) \| \| Scene 10.7 \| \| Scene 25.2 \| \| Scene 12.2 \| \| Scene 25.3 \| \| Scene 15.1 \| \| Scene 10.8 \| \| Scene 18.2 \| \| Scene 6.1 \| \| Scene 13.4 \| \| Scene 23.1 \| \| Scene 27.1 (Cue) \| \| Scene 22.3 \| \| Scene 7.2 \| \| Scene 21.2 \| \| Scene 13.2 (Cue) \| \| Scene 7.11 \| \|  \| |  |  |
| --- | --- | --- | --- | --- | --- | --- | --- | --- | --- | --- | --- | --- | --- | --- | --- | --- | --- | --- | --- | --- | --- | --- | --- | --- | --- | --- | --- | --- | --- | --- | --- | --- | --- | --- | --- | --- | --- | --- | --- | --- | --- | --- | --- | --- | --- | --- | --- | --- | --- | --- | --- | --- | --- | --- | --- | --- | --- | --- | --- | --- | --- | --- | --- | --- | --- | --- | --- | --- | --- | --- | --- | --- | --- | --- | --- | --- | --- | --- | --- | --- | --- | --- | --- | --- | --- | --- | --- | --- | --- | --- | --- | --- | --- | --- | --- | --- | --- | --- | --- | --- | --- | --- |
